# The Impact of the Coronavirus Pandemic on Vaccination Coverage in Latin America and the Caribbean

**DOI:** 10.3390/vaccines12050458

**Published:** 2024-04-25

**Authors:** Ignacio E. Castro-Aguirre, Dan Alvarez, Marcela Contreras, Silas P. Trumbo, Oscar J. Mujica, Daniel Salas Peraza, Martha Velandia-González

**Affiliations:** 1 Comprehensive Family Immunization Unit, Pan American Health Organization, Washington, DC 20037, USA; castroign@paho.org (I.E.C.-A.); alvarezdan@paho.org (D.A.);; 2 Department of Medicine, University of Central Florida College of Medicine, Orlando, FL 32827, USA; 3 Department of Evidence and Intelligence for Action in Health, Pan American Health Organization, Washington, DC 20037, USA; mujicaos@paho.org

**Keywords:** coronavirus pandemic, immunization coverage levels, diphtheria–tetanus–pertussis-containing vaccine, vaccination of newborns, zero-dose children, health disparities

## Abstract

Background: Routine vaccination coverage in Latin America and the Caribbean declined prior to and during the coronavirus pandemic. We assessed the pandemic’s impact on national coverage levels and analyzed whether financial and inequality indicators, immunization policies, and pandemic policies were associated with changes in national and regional coverage levels. Methodology: We compared first- and third-dose coverage of diphtheria–pertussis–tetanus-containing vaccine (DTPcv) with predicted coverages using time series forecast modeling for 39 LAC countries and territories. Data were from the PAHO/WHO/UNICEF Joint Reporting Form. A secondary analysis of factors hypothesized to affect coverages during the pandemic was also performed. Results: In total, 31 of 39 countries and territories (79%) had greater-than-predicted declines in DTPcv1 and DTPcv3 coverage during the pandemic, with 9 and 12 of these, respectively, falling outside the 95% confidence interval. Within-country income inequality (i.e., Gini coefficient) was associated with significant declines in DTPcv1 coverage, and cross-country income inequality was associated with declines in DTPcv1 and DTPcv3 coverages. Observed absolute and relative inequality gaps in DTPcv1 and DTPcv3 coverage between extreme country quintiles of income inequality (i.e., Q1 vs. Q5) were accentuated in 2021, as compared with the 2019 observed and 2021 predicted values. We also observed a trend between school closures and greater-than-predicted declines in DTPcv3 coverage that approached statistical significance (*p* = 0.06). Conclusion: The pandemic exposed vaccination inequities in LAC and significantly impacted coverage levels in many countries. New strategies are needed to reattain high coverage levels.

## 1. Introduction

**Figure 1:**
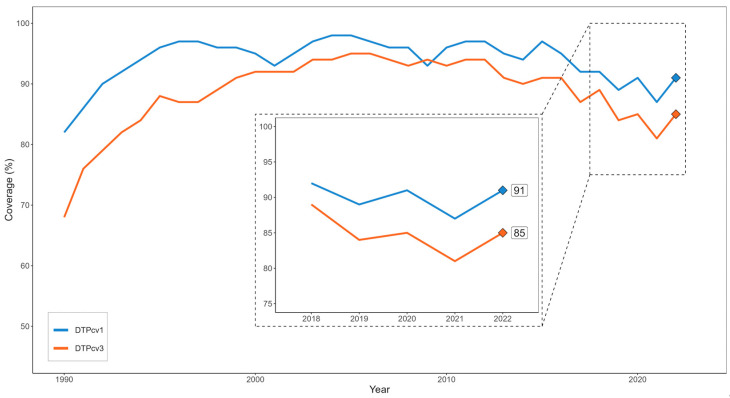
DTPcv1 and DTPcv3 coverage levels in Latin America and the Caribbean, 1990–2022.

Following the establishment of national immunization programs in the 1970s, countries in Latin America and the Caribbean (LAC) have made marked improvements in the control of vaccine-preventable diseases (VPDs) [1,2]. Over the last decade, however, third-dose coverage of diphtheria–pertussis–tetanus-containing vaccine (DTPcv3) has declined from 93% in 2010 to 84% in 2019, with rising numbers of children with incomplete schedules and those who have not received any doses (“zero-dose” children) (Figure 1) [1,3]. Decreases in DTPcv1 and DTPcv3 coverages have also been observed in other routine vaccines; in an analysis of coverage trends from 2015 to 2019, Plans-Rubió found that 10 out of 13 vaccines in the Americas decreased during this period [4]. Coverage declines have been pronounced among children living in poverty and other vulnerable situations, as well as those in hard-to-reach areas [5,6].

Causes of low vaccination coverage vary among countries but include health system factors (e.g., a lack of access to vaccination services), communication factors (e.g., vaccine hesitancy), and sociopolitical factors (e.g., poverty and political instability) [6]. The COVID-19 pandemic has exacerbated many of these problems. Containment measures; the suspension of school vaccination strategies and mass vaccination campaigns; the interruption of routine immunization services; the population’s reluctance to go to health centers due to fear of contracting the virus; and the diversion of resources for the coronavirus pandemic have resulted in the delayed administration of routine vaccines [7,8,9,10,11]. Additionally, the pandemic has politicized vaccination, worsening mistrust in health systems [12], coronavirus vaccines [13,14], and immunization services more generally [15]. As a result, coverages for DTP1cv1 and DTPcv3 in 2021 reached levels not seen in LAC since the early 1990s.

Against this backdrop, the Pan American Health Organization and World Health Organization (PAHO/WHO) have continued efforts to control and eliminate VPDs. Immunization Agenda 2030 (IA2030) and PAHO’s strategic document “Reinvigorating Immunization as a Public Good for Universal Health” provide concrete steps to aid countries in reaching all people, especially vulnerable populations and zero-dose children [1,16]. To support these goals, there is a need for an updated analysis of national coverage trends in LAC before and after the pandemic and the factors that may explain changes in coverage.

In this article, we estimate the effect of the COVID-19 pandemic on vaccination coverage for countries and territories in LAC. We then analyze whether national financial indicators, vaccine policies, and pandemic policies are associated with changes in coverage levels and cross-country distributive inequality. We conclude by discussing the causes of declining coverage rates and propose strategies to reverse these declines.

## 2. Methodology

PAHO/WHO and UNICEF publish annual vaccination coverages based on country reports collected through the Joint Reporting Form (JRF) [3]. We performed an analysis of these data in LAC from 1990 to 2022 [3]. Of the 49 countries and territories in LAC, 39 were included in the analysis, accounting for 99% of the region’s population [3].

We chose first-dose coverage of diphtheria–tetanus–pertussis-containing vaccine in children aged <12 months (DTPcv1) as an indicator of access to health services and third-dose coverage (DTPcv3) as an indicator of immunization program follow-up and performance. Consistent with IA2030, “zero-dose” children—i.e., those not receiving any dose of DTPcv before age <12 months—were considered to have limited access to health services [16].

As an initial analysis, we compared absolute coverage changes in DTPcv1 and DTPcv3 between 2019 and 2021. To distinguish between pre-pandemic trends in vaccination coverage and the impact of the pandemic on DTPcv1 and DTPcv3 coverages, we then used time series forecast modeling to compare predicted coverages (i.e., those expected if a pandemic had not occurred) with country-reported coverage levels in 2021. DTPcv1 and DTPcv3 coverages from 1990 to 2019 were used in the forecast model. Although concerns about COVID-19 began in late 2019, countries did not implement significant pandemic measures until 2020. As such, we defined all years before 2020 as pre-pandemic. Predicted coverages were estimated through Holt’s linear trend model, which aims to describe the behavior of a trending time series [17].

We performed a secondary analysis of factors hypothesized to affect coverages during the pandemic, comparing observed to predicted coverage changes for DTPcv1 and DTPcv3 in 2021. Supplemental Table S1 outlines the data source, categories, methodology, and statistical test used for each variable [3,18,19,20,21,22]. Furthermore, we conducted an exploratory analysis of vaccination coverage inequalities across countries ranked by their mean income per capita (deflated and purchase-power-adjusted), calculating standard summary measures of health inequality, including Kuznets-like inequality gaps and the slope index of inequality (SII) through a log-linear weighted regression model, as described elsewhere [22,23].

We analyzed data with Microsoft Excel and the R statistical software (version 4.3.2) [24]. Time series modeling was performed using the fpp2 package (version 2.5) [17]. During one phase of the article’s development, generative AI was used for general editing and to draft a preliminary version of the discussion section (which was then significantly modified); however, the technology was not subsequently utilized, and the content presented is the creation and responsibility of the authors. 

## 3. Results

From 2019 to 2021, 30 of 39 countries and territories had absolute declines in DTPcv1 coverage, with 13 declining <5 percentage points (%pt), 12 declining 5–10 %pt, and five declining >10 %pt (Supplemental Table S2). Four countries and territories reported increased DTPcv1 coverage during the pandemic, most notably Anguilla (79% in 2019 vs. 88% in 2021). During the same period, 32 of 39 countries experienced decreases in DTPcv3 coverage, with 13 declining <5 %pt, 12 declining 5–10 %pt, and 7 declining >10 %pt. Haiti reported the greatest increase in DTPcv3 coverage (66% in 2019 vs. 73% in 2021) during the pandemic.

**Figure 2:**
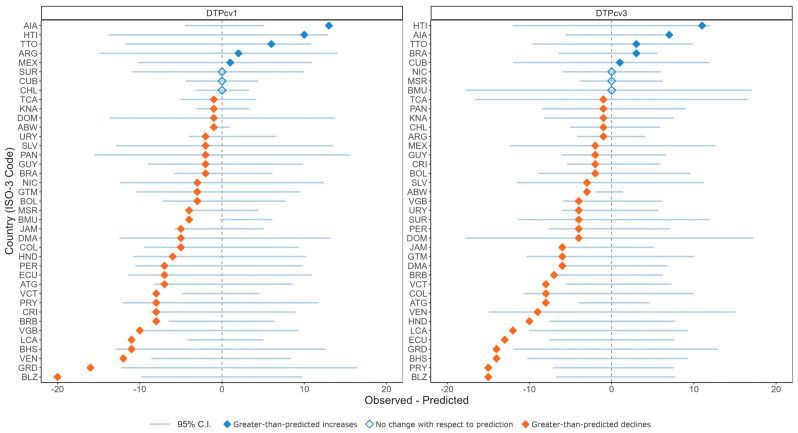
Observed versus predicted changes in DTPcv1 and DTPcv3 coverage in 2021 in Latin America and the Caribbean, 39 countries and territories with available data.

For 2021, time series forecast modeling showed that 31 countries and territories experienced greater-than-predicted declines in DTPcv1 coverage, with 9 falling outside the 95% confidence interval (CI) (Figure 2). For DTPcv3 coverage, 31 countries and territories experienced greater-than-predicted declines; 12 of these were outside the CI. Coverage in Belize for DPTcv1, for example, decreased by 20 %pt with respect to the prediction’s mean, falling outside the CI and suggesting that the decline may be due to the pandemic.

Figure 2 compares observed to predicted changes in coverage for DTPcv1 and DTPcv3 and, thus, a country or territory’s access to vaccines (DTPcv1) and to follow-up immunization services (DTPcv3). In 27 of 39 countries and territories, access and follow-up to immunization services both worsened. But some countries did not follow this pattern. For example, in Mexico, follow-up worsened by 2 %pt more than predicted, but access improved by 1% more than predicted. Conversely, in Brazil, follow-up improved by 3 %pt more than predicted, but access worsened by 2 %pt.

A secondary analysis of explanatory variables showed that vaccine administration in schools and national pandemic policies regarding public transportation and stay-at-home orders were not associated with statistically significant changes in DTPcv1 or DTPCv3 coverages at the regional level (Table 1). We observed a trend between school closures and greater-than-predicted declines in DTPcv3 coverage that did not reach statistical significance (*p* = 0.06). Of the three financial indicators evaluated, only the Gini index of income inequality was statistically significant at the regional level. Countries with greater income inequality had greater DTPcv1 coverage declines than countries with less income inequality (−5 %pt vs. −1.5 %pt, *p* = 0.04).

An exploratory analysis of DTPcv1 and DTPcv3 coverage inequalities showed the effect of income inequality *between* countries on their magnitude and trends at the regional level (Table 2). When ranking countries from poorest to richest by their mean GDP per capita, those in the poorest quintile fared consistently worse than those in the richest quintile; indeed, in 2021, the observed absolute and relative inequality gaps in DTPcv1 and DTPcv3 coverage between these extreme quintiles were larger than predicted.

**Figure 3:**
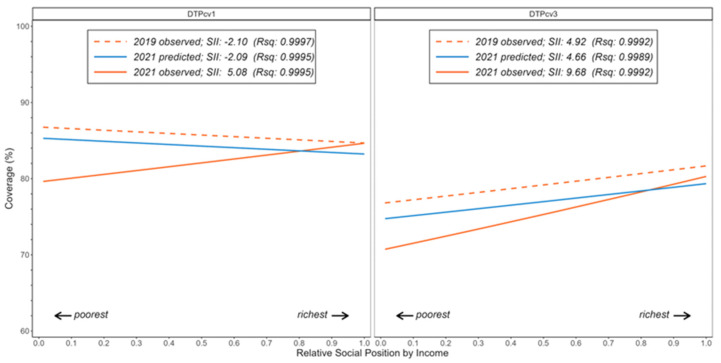
Income-related cross-country inequality gradients in DTPcv1 and DTPcv3 coverage, Latin America and the Caribbean, 2019 and 2021.

Moreover, there were clear regional inequality gradients in DTPcv1 and DTPcv3 coverage that were evident across the social hierarchy (“social hierarchy” refers to the cross-country gradient formed when countries are ranked by GDP per capita from poorest to richest) as defined by country GDP per capita (Table 2). The pro-poor inequality pattern in DTPcv1 coverage, initially observed in 2019 (and predicted by 2021), was inverted in 2021; the SII increased from −2.10 %pt DTPcv1 in 2019 to 5.08 %pt in 2021. The pro-rich inequality pattern in DTPcv3 coverage became more evident between 2019 and 2021, with the SII rising from 4.92 %pt in 2019 to 9.68 %pt in 2021 (Figure 3).

## 4. Discussion

We found that routine vaccination coverage in most LAC countries and territories declined during the COVID-19 pandemic. While only 9 and 12 LAC countries and territories, respectively, experienced statistically significant greater-than-predicted declines in DTPcv1 and DTPcv3 coverage, there was a clear trend toward lower-than-predicted coverages. For both DTPcv1 and DTPcv3, 79% of countries had larger-than-expected declines in coverage based on the pattern observed over the last 30 years.

Large studies have shown that the coronavirus pandemic negatively impacted routine vaccination rates in countries around the world [25,26,27,28]. Consistent with previous research, the decrease in coverage in LAC countries and territories likely stems from service disruptions, a lack of vaccine availability, the population’s fear of visiting health centers, and the diversion of resources to pandemic-related activities [25,26,27,28,29,30]. This being acknowledged, the fact that eight LAC countries and territories had no decreases in DPTcv3 coverage suggests that some countries may have been able to quickly recover from service disruptions in early 2020 due to their strong immunization programs [25], relatively mild income inequality, or other factors. Further research, potentially in the form of country case studies, could explore what factors were associated with immunization program resilience during the pandemic.

We found income inequality to be associated with declines in DTPcv1 coverage during the pandemic. Previous research has shown that lower-middle-income countries (LMICs) experienced greater declines than high-income countries (HICs) during the pandemic [25]. Similarly, we observed a significant slope index of inequality in DTPcv1 and DTPcv3 coverages across the cross-country gradient defined by GDP per capita (i.e., a measure of *between-country* income inequality), a pro-rich slope that was steeper in 2021. Furthermore, we found that *within-country* income inequality, as measured by the Gini index, predicted greater-than-expected coverage declines, aligning with other reports highlighting how the pandemic revealed and deepened existing health inequities [31,32,33]. Our research also revealed a possible association between school closures and greater-than-predicted declines in DTPcv3 coverage, although statistical significance was not reached (*p* = 0.06).

Previous research has shown that DTPcv1 and DTPCv3 coverages in the Americas declined by 3.0% and 2.7%, respectively, from 2015 to 2019 [4]. We found that coverage levels rebounded to pre-pandemic levels in 2022 but remained well below 2015 levels. The improvement suggests that LAC countries can reverse the decline. Still, the overall trend remains concerning, and new challenges have emerged in the wake of the COVID-19 pandemic. For example, the politicization of the pandemic has created misinformation on vaccines [13], and the pandemic may have decreased the public’s confidence in immunization programs [25]. Amid this complexity, the pandemic may serve as a “wake-up call” for the region to re-prioritize immunization and re-evaluate the strategies needed to ensure high and homogenous coverages.

Recommendations based on our findings, a review of the literature, and expert opinion include addressing vaccine hesitancy, targeting socioeconomic factors driving undervaccination, and focusing on reducing the number of zero-dose children while avoiding unintended negative impacts on follow-up schedules [1,34,35]. Vaccine hesitancy in LAC is multifactorial, likely to vary among and within countries, and is arguably the largest factor affecting coverages [6]. Using the Strategic Advisory Group of Experts (SAGE) working group’s framework for vaccine hesitancy, Guzman-Holst found that hesitancy in the region centered on individual/group influences (e.g., safety misconceptions), contextual influences (e.g., low socioeconomic status), and vaccine and vaccination-specific issues (e.g., negative experiences at health centers) [6,36]. Countries should identify specific causes of vaccine hesitancy at the local level, develop strategies to combat misinformation, and educate healthcare workers who themselves may be hesitant toward vaccines [1,14].

Countries must also develop contingency plans to reach children who missed vaccines and to minimize disruptions during future public health emergencies [13]. Strategies, such as campaigns and periodic outreach activities, must be in place to maintain routine immunization in schools and integrate immunization with all essential health services across primary healthcare. This is particularly important in areas with significant income inequality, since lockdowns may disproportionately affect the most vulnerable and economically disadvantaged children [31].

Finally, the decrease in coverage due to the pandemic may lead to an increase in the prevalence of VPDs. This may be especially true for diseases with high transmissibility rates, such as measles, where a decrease in coverage can result in increased prevalence in the short term [37]. The pandemic highlights an opportunity for countries to reaffirm their commitment to immunization and make the investments needed to increase coverage. Early results suggest that countries may already be making such an investment. In April 2023, Caribbean countries and territories signed the Declaration of Nassau to strengthen national immunization programs [38]. In Brazil, the president championed a national movement in favor of vaccination [39], and the senate held sessions to discuss vaccination as a public good [40]. Meanwhile, Argentina held a very successful Vaccination Week, resulting in more than 1.1 million vaccines being administered and culminating with a community event called “El Festival de las Vacunas” [41]. Such post-pandemic activities and commitments are vital to raising coverage levels.

The principal strengths of this study include the use of time series modeling to differentiate pre-existing coverage trends from changes in coverage that might be attributed to the pandemic and a robust secondary analysis of the role of within- and between-country inequality in vaccination outcomes. However, this study has several notable limitations. Data submitted by countries to the JRF are administrative and subject to occasional issues in quality and recording (e.g., outdated census data) [42,43]. Although the PAHO/WHO and UNICEF strive to address these concerns by triangulating administrative data with coverage surveys, resulting coverage adjustments may be inaccurate. Additionally, our model assumed a linear trend in coverage patterns, but some countries and territories may have seasonal patterns, thereby limiting our model’s accuracy. It is also worth noting that the pandemic may have caused fluctuations in monthly vaccination coverages not detected in our model. Lastly, LAC includes many countries and territories with small populations; in these cases, minor changes in coverages may not be statistically significant but still meaningfully impact patients. We have accordingly argued that it is important to consider both trends and statistical significance when evaluating coverage changes following the pandemic.

Our secondary analysis was constrained by limited data on factors that may have affected vaccination coverage during the pandemic. We attempted to incorporate factors like “trust in science” and “trust in the government” that were included in other studies on changes in vaccination coverage during the pandemic [18,44]. Unfortunately, only a small proportion of countries in LAC had available data for these factors. A valuable area of future research would be the creation of a multivariable model to explain changes in coverage levels that incorporates the factors in our secondary analysis, as well as others related to confidence in the health system, government, and immunization programs [45].

## 5. Conclusions

The COVID-19 pandemic exposed vaccination inequities in LAC and significantly impacted coverage levels in specific countries and territories. Although coverage levels continued to decrease during the pandemic, they have rebounded to pre-pandemic levels. This signals an opportunity for continued improvement and suggests that the pandemic may have prompted a renewed investment in immunization in the Americas. Strategies to reattain and maintain high coverage include conducting outreach activities for marginalized groups, targeting socioeconomic factors driving vaccination inequities, employing a multifaceted approach to confront vaccine hesitancy, reaffirming national commitment to vaccination, and developing plans to minimize disruptions in future public health emergencies [1,13,34,35].

## Figures and Tables

**Table 1:** Factors associated with changes in observed versus predicted DTPcv1 and DTPcv3 coverages during the coronavirus pandemic, 2019–2022.

	DTPcv 1		DTPcv3	
Variable	Median (IQR)	*p*-Value	Median (IQR)	*p*-Value
**Vaccine administration in school**				
Yes	−4.00 (−8.00; −1.00)	0.98	−3.00 (−8.00; −1.00)	0.55
No	−3.00 (−6.00; −1.50)		−4.00 (−7.50; −1.50)	
**School-closing policies**				
None/recommended	−2.50 (−3.75; −1.25)	0.37	−1.50 (−3.75; 0.00)	0.06
Required	−3.00 (−7.25; −0.75)		−4.00 (−8.25; −1.75)	
**Stay-at-home policies**				
None/recommended	−3.00 (−5.00; −2.00)	0.60	−3.00 (−6.00; −2.00)	0.82
Required	−3.00 (−7.00; 0.00)		−4.00 (−8.00; −1.00)	
**Closing of public transportation**				
None	−3.00 (−5.00; −1.00)	0.95	−2.00 (−4.00; −1.00)	0.40
Recommended/required	−3.00 (−7.00; −1.00)		−4.00 (−8.00; −1.00)	
**WB income group**				
High	−2.00 (−7.25; −1.00)	0.57	−2.00 (−4.75; −1.00)	0.41
Middle	−4.00 (−7.25; −1.75)		−4.00 (−8.50; −2.00)	
**GDP per capita**				
High	−1.85 (−4.00; −1.00)	0.18	−2.08 (−4.00; 0.00)	0.14
Middle	−4.85 (−8.00; −1.00)		−5.15 (−8.00; −2.00)	
Low	−5.08 (−7.00; −3.00)		−5.46 (−10.00; −2.00)	
**GINI index**				
Less unequal	−1.50 (−2.75; 0.75)	0.04 *	−2.50 (−4.00; −1.25)	0.54
More unequal	−5.00 (−7.00; −3.00)		−6.00 (−10.00; −1.00)	
**SDIx 2021**				
High	−2.00 (−7.00; 0.00)	0.42	−1.00 (−7.00; −1.00)	0.65
Middle	−6.00 (−8.00; −1.00)		−4.00 (−8.25; −2.00)	
Low	−4.00 (−6.25; −2.75)		−6.00 (−10.50; −1.50)	

IQR: interquartile range; WB: World Bank; GDP: gross domestic product; SDIx: PAHO Sustainable Development Index; * *p*-Value < 0.05.

**Table 2:** DTPcv1 and DTPcv3 vaccine coverage by country quintiles of income per capita, regional average, and Kuznets-like inequality gap metrics. Latin America and the Caribbean, 2019 and 2021.

Vaccine	Timepoint, Scenario	Vaccine Coverage (%)						Q1 v Q5 Inequality Gap	
		Q1	Q2	Q3	Q4	Q5	Setting Average	Absolute Gap	Relative Gap
DTPcv1	2019 observed	86.9	91.9	82.5	87.3	91.5	86.0	−4.6	0.95
	2021 predicted	86.2	85.8	82.6	92.4	89.8	84.7	−3.6	0.96
	2021 observed	82.4	80.9	80.6	93.1	91.9	82.5	−9.5	0.90
DTPcv3	2019 observed	76.7	87.1	75.3	84.0	92.4	79.8	−15.7	0.83
	2021 predicted	75.5	82.9	75.7	86.8	90.9	77.8	−15.4	0.83
	2021 observed	72.8	73.6	75.1	85.1	90.3	75.9	−17.5	0.81

Notes: Q1 = poorest; Q3 = median; Q5 = richest.

## Data Availability

Data are contained within the article and also presented in Supplemental Table S2.

## Supplementary Materials

The following supporting information can be downloaded at: https://www.mdpi.com/article/10.3390/vaccines12050458/s1. Supplemental Table S1 (Data sources and methodology for factors hypothesized to be associated with changes in DTPcv1 and DTPcv3 coverage rates during the coronavirus pandemic, 2019–2021) and Supplemental Table S2 (“Data dictionary” and “Observed versus predicted changes in DTPcv1 and DTPcv3 coverage and associated variables, Latin America and the Caribbean, 2019 and 2021).
