# Supplementary material for: The Impact of the Coronavirus Pandemic on Vaccination Coverage in Latin America and the Caribbean
Source: Vaccines (Basel). 2024 Apr 25;12(5):458. doi: 10.3390/vaccines12050458 (PMC11125655; doi:10.3390/vaccines12050458)
Supplement: Supplementary file 1 [file vaccines-12-00458-s001.zip › Supplemental table S1.pdf]

Supplemental table S1: Data sources and methodology for factors hypothesized to be associated with changes in DTPcv1 and DTPcv3 coverage rates during the coronavirus pandemic, 2019-2021

Variable	Data source	Categories	Methodology	Statistical test
Vaccine administration in schools	PAHO/WHO/UNICEF Joint Reporting Form [3]	1. Yes<br>2. No	Responses to the question "Are any routine doses of vaccines on the national immunization schedule regularly delivered to children at school?" in 2021 were analyzed.	DTPcv1: t-test<br>DTPcv3: t-test
School closing policies	Oxford University COVID Policy Tracker [18]	1. None or recommended<br>2. Required	Countries were classified as none, recommended, or required based on the number of days in which restrictions were in place in 2020 and 2021. Countries were then divided into two comparable groups.	DTPcv1: mw-test<br>DTPcv3: t-test
Stay-at-home policies	Oxford University COVID Policy Tracker [18]	1 None or recommended<br>2 Required	Countries were classified as none, recommended, or required based on the number of days in which restrictions were in place in 2020 and 2021. Countries were then divided into two comparable groups.	DTPcv1: t-test<br>DTPcv3: t-test
Closing of public transport	Oxford University COVID Policy Tracker [18]	1. None<br>2. Recommended or required	Countries were classified as none, recommended, or required based on the number of days in which restrictions were in place in 2020 and 2021. Countries were then divided into two comparable groups.	DTPcv1: t-test<br>DTPcv3: mw-test
Income group	World Bank [19]	1. High-income<br>2. Middle-income	Countries were divided into two groups using the World Bank's 2021 classification. High-income countries were classified as high-income. Middle-low and middle-high income countries were classified as middle-income.	DTPcv1: t-test<br>DTPcv3: t-test
Gross domestic product per capita	Global Health Data Exchange [20]	1. High<br>2. Middle<br>3. Low	Countries were divided into three groups by terciles.	DTPcv1: ANOVA<br>DTPcv3: ANOVA
Gini index	World Bank [21]	1. Less unequal<br>2. More unequal	Countries were divided into two groups by the median.	DTPcv1: t-test<br>DTPcv3: mw-test
Sustainable Development Index (SDIx) 2021*	PAHO SDIx [22]	1. High<br>2. Middle<br>3. Low	Countries were divided into three groups by terciles.	DTPcv1: ANOVA<br>DTPcv3: ANOVA

Mw-test: Mann–Whitney–Wilcoxon test.
*Data not publicly available.
