# Supplementary material for: The Impact of the Coronavirus Pandemic on Vaccination Coverage in Latin America and the Caribbean
Source: Vaccines (Basel). 2024 Apr 25;12(5):458. doi: 10.3390/vaccines12050458 (PMC11125655; doi:10.3390/vaccines12050458)
Supplement: Supplementary file 1 [file vaccines-12-00458-s001.zip › supplementary_table_S2_data_dictionary.pdf]

Supplementary Table S2 – Data Dictionary

Columns:

1. ISO_CODE: The ISO 3166-1 alpha-3 codes. These are three-letter country codes defined in ISO 3166-1.
2. COUNTRY_NAME: The official country or territory name in English.
3. DTP1_2019: The official coverage for each country or territory in 2019.
4. DTP1_2021: The official coverage for each country or territory in 2021.
5. DTP1_2021_PRED: The predicted coverage for the country or territory in 2021, computed using all available historical data up to 2019 (including).
6. CHANGE_DTP1: The difference between the official coverage in 2021 and the official coverage in 2019.
7. CHANGE_DTP1_PRED: The difference between the predicted coverage for 2021 and the official coverage in 2021.
8. DTP3_2019: Same as DTP1_2019, but for DTP3.
9. DTP3_2021: Same as DTP1_2021, but for DTP3.
10. DTP3_2021_PRED: Same as DTP1_2021_PRED, but for DTP3.
11. CHANGE_DTP3: Same as CHANGE_DTP1, but for DTP3.
12. CHANGE_DTP3_PRED: Same as CHANGE_DTP1_PRED, but for DTP3.
13. WB: The World Bank 2022-2023 Country Classification by Income Level for each country or territory.
14. IHME_GDP: GDP per capita (constant, purchasing power parity), 2021, IHME estimates, for countries or territories included in this manuscript.
15. SCHOOL_CLOSING: Based on the “School closing” variable of the Oxford COVID Policy Tracker dataset. See codebook for more information.
16. CLOSE_PUBLIC_TRANSPORT: Based on the “Close public transport” variable of the Oxford COVID Policy Tracker dataset. See codebook for more information.
17. STAY_AT_HOME: Based on the “Stay at home requirements” variable of the Oxford COVID Policy Tracker dataset. See codebook for more information.
18. SCHOOL_VAX_STATUS: From the PAHO/WHO/UNICEF Joint Reporting Forum, responses to the question “Are any routine doses of vaccines on the national immunization schedule regularly delivered to children at school?” in 2021 were analyzed and classified into “Yes” and “No”.
19. GINI_INDEX_N_TILE: Gini index for countries or territories (included in this manuscript) where the most recent evaluation was no older than 2010 (including).
20. SDI_N_TILE: The PAHO sustainable development index (SDI) for 2021, for countries or territories included in this manuscript.
